# What Shall I Teach My Child?

**Published:** 1912-12

**Authors:** 


### What Shall I Teach My Child?

This question is the one most often found on the lips of every one that has come in touch with the new movement of race betterment. The facts of the venereal peril burns heavily in the heart of the anxious parent. Feeling the wrong done them by not having been properly instructed by their parents, the fathers and mothers of the present generation mean to correct this stupendous error. They are asking for information from every source, and, to meet this demand, there will be given practical suggestion on this question in this department, beginning with the January number.

